# Supplementary material for: Antiviral Evaluation of UV-4B and Interferon-Alpha Combination Regimens against Dengue Virus
Source: Viruses. 2021 Apr 27;13(5):771. doi: 10.3390/v13050771 (PMC8145572; doi:10.3390/v13050771)
Supplement: Supplementary file 1 [file viruses-13-00771-s001.zip › viruses-1196427-supplementary.pdf]

viruses

Article

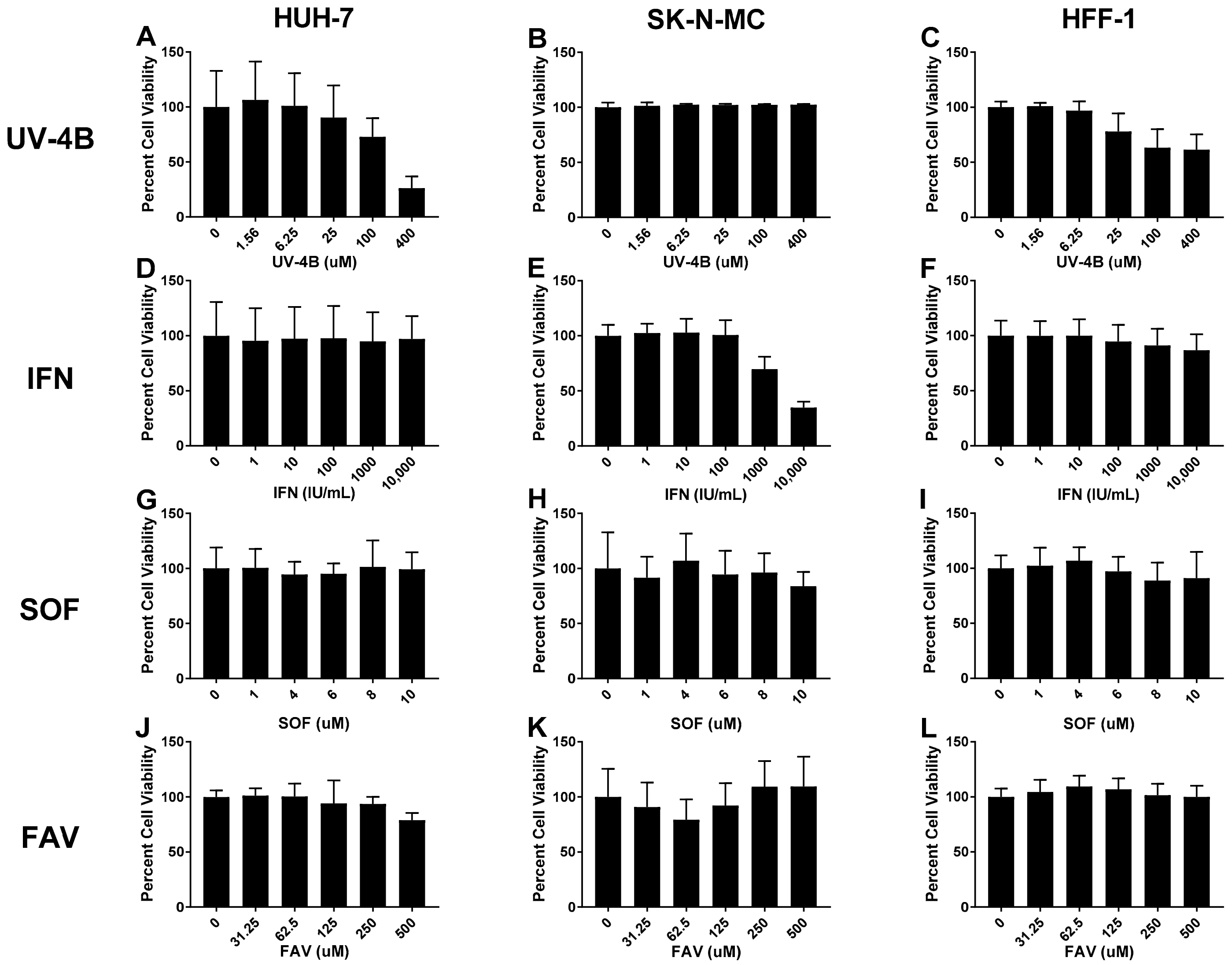

Supplementary Figure 1. The effect of UV-4B (A-C), interferon-alpha (IFN; D-F), sofosbuvir (SOF; G-I), and favipiravir (FAV; J-L) on the viability of uninfected HUH-7, SK-N-MC, and HFF-1 cell lines. Cell viability was measured after three days of drug exposure using the commercially available WST-1 assay kit as per the manufacturer's instructions. Cell viability is reported as percent cell viability relative to an untreated control. Columns represent the mean of 6 independent samples and error bars correspond to one standard deviation.
